# Changes in H3K27ac following lipopolysaccharide stimulation of nasopharyngeal epithelial cells

**DOI:** 10.1186/s12864-018-5295-4

**Published:** 2018-12-27

**Authors:** Lisa Borghini, Martin Hibberd, Sonia Davila

**Affiliations:** 1 Human Genetics, Genome Institute of Singapore, Singapore, 138672 Singapore; 2 Infectious Disease, Genome Institute of Singapore, Singapore, 138672 Singapore; 3 Present Address: Pathogen Molecular Biology, Infectious & Tropical Disease, London School of Hygiene & Tropical Medicine, London, WC1E 7HT UK; 4 Present Address: SingHealth Duke-NUS Institute of Precision Medicine (PRISM), Singapore, 169609 Singapore

**Keywords:** H3K27ac, Enhancers, LPS, Epithelial cells, RELA

## Abstract

**Background:**

The epithelium is the first line of defense against pathogens. Notably the epithelial cells lining the respiratory track are crucial in sensing airborne microbes and mounting an effective immune response via the expression of target genes such as cytokines and chemokines. Gene expression regulation following microbial recognition is partly regulated by chromatin re-organization and has been described in immune cells but data from epithelial cells is not as detailed. Here, we report genome-wide changes of the H3K27ac mark, characteristic of activated enhancers and promoters, after stimulation of nasopharyngeal epithelial cells with the bacterial endotoxin Lipopolysaccharide (LPS).

**Results:**

In this study, we have identified 626 regions where the H3K27ac mark showed reproducible increase following LPS induction in epithelial cells. This indicated that sensing of LPS led to opening of the chromatin in our system. Moreover, this phenomenon seemed to happen extensively at enhancers regions and we could observe instances of Super-enhancer formation. As expected, LPS-increased H3K27ac regions were found in the vicinity of genes relevant for LPS response and these changes correlated with up-regulation of their expression. In addition, we found the induction of H3K27ac mark to overlap with the binding of one of the NF-kB members and key regulator of the innate immune response, RELA, following LPS sensing. Indeed, inhibiting the NF-kB pathway abolished the deposition of H3K27ac at the *TNF* locus, a target of RELA, suggesting that these two phenomena are associated.

**Conclusions:**

Enhancers’ selection and activation following microbial or inflammatory stimuli has been described previously and shown to be mediated via the NF-kB pathway. Here, we demonstrate that this is also likely to occur in the case of LPS-sensing by nasopharyngeal epithelial cells as well. In addition to validating previous findings, we generated a valuable data set relevant to the host immune response to epithelial cell colonizing or infecting pathogens.

**Electronic supplementary material:**

The online version of this article (10.1186/s12864-018-5295-4) contains supplementary material, which is available to authorized users.

## Background

The epithelium constitutes a natural barrier against pathogens and is particularly important in fighting infection. Indeed, epithelial cells are the first to detect microbes and are crucial in mounting an effective innate immune response as well as interacting with more specialized immune cells to initiate the adaptive immunity processes [1]. Especially, the epithelial cells in the respiratory tract are critical in regards to defense against pathogens entering the body through inhalation [2].

These cells recognize specific microbial components through pattern recognition receptors, mainly Toll-like receptors (TLR) [3]. Upon recognition, signaling pathways are activated and lead to the expression regulation of genes such as cytokines, chemokines and antimicrobials by transcription factors [4]. Regulation by transcription factors goes together with chromatin organization which helps coordinate gene expression. Epigenetic changes following stimulation has received a vast interest recently and was found to be determined by the cell type targeted as well as the environmental signals applied. Indeed, selection of regulatory regions, particularly enhancers, following stimulation consists of interplay between lineage-determining and signal-dependent transcription factors that together achieve specific cell response to a particular stimulus [5].

Among the various changes and marks added onto histone proteins following re-organization after stimulation, Histone 3 Lysine 27 acetylation (H3K27ac) seems to be very dynamic and has been shown to be important in regulation of the immune response [6, 7]. This mark indicates active enhancers and promoters [8].

The changes in chromatin following microbial stimuli has been mostly studied in immune cells, most often in mouse macrophages [7, 9, 10]. Here, we studied a human nasopharyngeal epithelial cell line, Detroit 562 cells, used extensively in studying airborne infectious diseases such as infection with *Neisseria meningitidis* [11], *Streptococcus pneumoniae* [12] and influenza virus [13] among others. We challenged these cells with Lipopolysaccharide (LPS), a potent gram negative bacterial endotoxin that targets TLR4, and described the changes in H3K27ac mark induced by the stimulation.

We have recently investigated the response of one of the master regulator of innate immunity, Nuclear Factor kappa B (NF-kB) member RELA, to the same stimulus and in the same cell line [14]. Thus, we were able to examine LPS-induced H3K27ac regions identified with regards to binding of RELA, one of the NF-kB member activated downstream of TLR4. This factor has been previously shown to be crucial for selecting enhancers and recruiting co-factors following stimulation [15, 16].

The data set described here characterizes the response to LPS by epithelial cells. LPS is commonly used as a model for bacterial infection and nasopharyngeal epithelial cells are particularly relevant for airborne pathogen infection. Thus, this study may provide insights into the host response to bacteria responsible for infectious respiratory diseases.

## Results

**Fig. 1:**
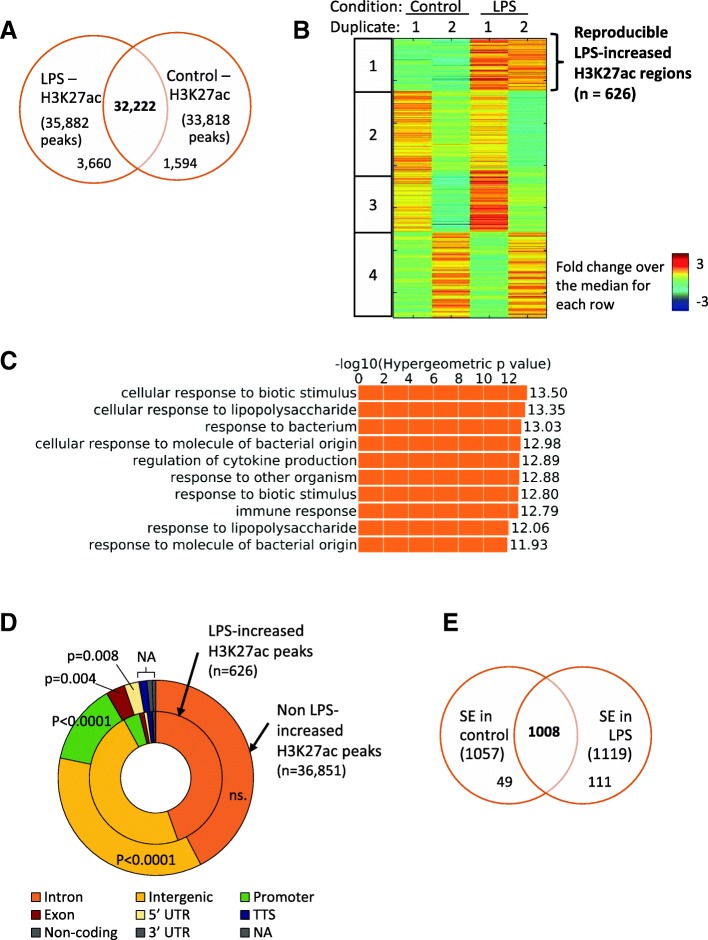
Identification and characterization of LPS-induced H3K27ac peaks. **a**: Overlap of H3K27ac peaks in Control and LPS condition. ChIP-seq experiment for H3K27ac was performed after treating Detroit 562 cells with LPS at 1μg/ml or fresh medium (Control) for 100 min. Final reproducible peaks identified in each condition were compared, two peaks were considered overlapping when their summit was located less than 1000 bp apart. **b**: Identification of LPS-increased H3K27ac peaks. H3K27ac ChIP-seq data from two replicates under no (Control) or LPS treatment were used to measure the signal intensity and perform differential binding analysis. Differential peaks were further clustered into 4 groups. **c**: Gene ontology analysis. LPS-increased peaks were associated to their single closest gene and Gene Ontology analysis was performed using all H3K27ac peaks identified as background. Top 10 biological processes terms are reported. D: Annotation of H3K27ac regions. LPS-increased (inside ring) and non LPS-increased (outside ring) H3K27ac peaks were assigned to the genomic feature they were located into. Proportion of each feature are represented. Comparison between the two sets of peaks was performed using a Chi-square test and the pearson *P*-values are reported. E: Overlap of Super-Enhancers. Super-enhancers (SE) identified in LPS and Control conditions were compared, two peaks were considered overlapping if they have at least 1 bp in common

In order to investigate changes in chromatin after LPS sensing by epithelial cells, we performed ChIP-seq experiments for H3K27ac with or without treatment, in Detroit 562 cells. We identified 35,882 and 33,818 peaks under “no treatment” (Control) and LPS stimulation respectively (Additional file 1). The two sets of peaks were greatly overlapping with 32,222 common peaks (Fig. 1a) (out of 37,476 total peaks, 86%). Differential binding analysis allowed us to determine LPS-induced changes in H3K27ac. 626 peaks were identified as LPS-increased – peaks for which the signal after LPS stimulation was higher compared to the control – in both ChIP-seq replicates (Group 1) while other groups of peaks showed less reproducible changes (Groups 2, 3 and 4) (Fig. 1b). No regions were found to have a lower H3K27ac signal after LPS treatment than before. The prevalence of increased H3K27ac mark rather than decreased was also observed when comparing the log2 fold change (log2FC) in signal between LPS and Control. The top 300 increased peaks had a median log2FC of 1.078 while the bottom 300 decreased peaks showed a median log2FC of 0.602 only (Additional file 2 A). This shows that LPS stimulation in our system induces chromatin opening rather than closing.

Expectedly, Gene Ontology analysis on the genes assigned to LPS-increased H3K27ac regions revealed enrichment for relevant biological processes terms such as “cellular response to Lipopolysaccharide” (−log10 *P*-value = 13.35) or “response to bacterium” (-log10P-value = 13.03), coming up as some of the top hits (Fig. 1c and Additional file 3 for all terms). These results confirm that the LPS-regulated H3K27ac regions identified are reliable and consistent with the stimulus applied.

Next, we annotated the H3K27ac peaks identified to investigate whether they were more likely to lie in specific genomic regions. When looking at all peaks (found in Control and/or LPS condition), most were found within Intronic (42.4%), Intergenic (36.1%) and Promoter (13.2%) regions, consistent with H3K27ac being a mark of active promoters and enhancers [8]. While LPS-increased peaks also showed the same trend with 44.6% of Intronic, 47.4% of Intergenic and 4.0% of Promoter peaks, there was significantly more intergenic (*p* < 0.0001) and less Promoter (p < 0.0001) peaks compared to non LPS-increased H3K27ac regions. In addition, there was also less exonic (*p* = 0.004) and 5’UTR (*p* = 0.008) peaks (Fig. 1d). This observation is consistent with other reports showing a crucial role for enhancers in cellular response to stimulus [5]. This was further supported when looking at the distribution of enhancers and promoters along the H3K27ac regions ordered according to their variation in signal after LPS stimulation. Promoters were rarer among the 300 increased and decreased peaks compared to the unchanged H3K27ac peaks while enhancers showed the opposite trend (Additional file 4 B and Additional file 2 B), suggesting that promoters might be less relevant in regulating the response to LPS but necessary to regulate constitutive cell function which do not vary under stimulation.

In addition, we performed super-enhancers analysis as it was shown previously that stimulation can re-organize cellular super-enhancers [15]. Using H3K27ac ChIP-seq signal, we identified 1057 and 1119 super-enhancers in Control and LPS condition respectively (Additional file 5 A). As previously shown, these super-enhancers were much bigger than other enhancers (Additional file 5 B). Gene Ontology analysis revealed relevant biological processes such as “immune system process” (Raw *P*-value = 2.7e-22) or “regulation of apoptotic process” (Raw P-value = 1.7e-18) as top terms for the LPS super-enhancers that were not present in the top Gene Ontology results for Control super-enhancers (Additional file 5 C). Moreover, we found 111 super-enhancers present in LPS condition but not in Control, while 49 super-enhancers were exclusively found in Control (Fig. 1e). One of the gained super-enhancers after LPS stimulation occurred upstream of the *CSF2* locus (Additional file 5 D) which encodes a cytokine activating macrophages and dendritic cells [17]. These results suggest that LPS stimulation not only opens chromatin but also induces formation of super-enhancers around certain immune genes.

**Fig. 2:**
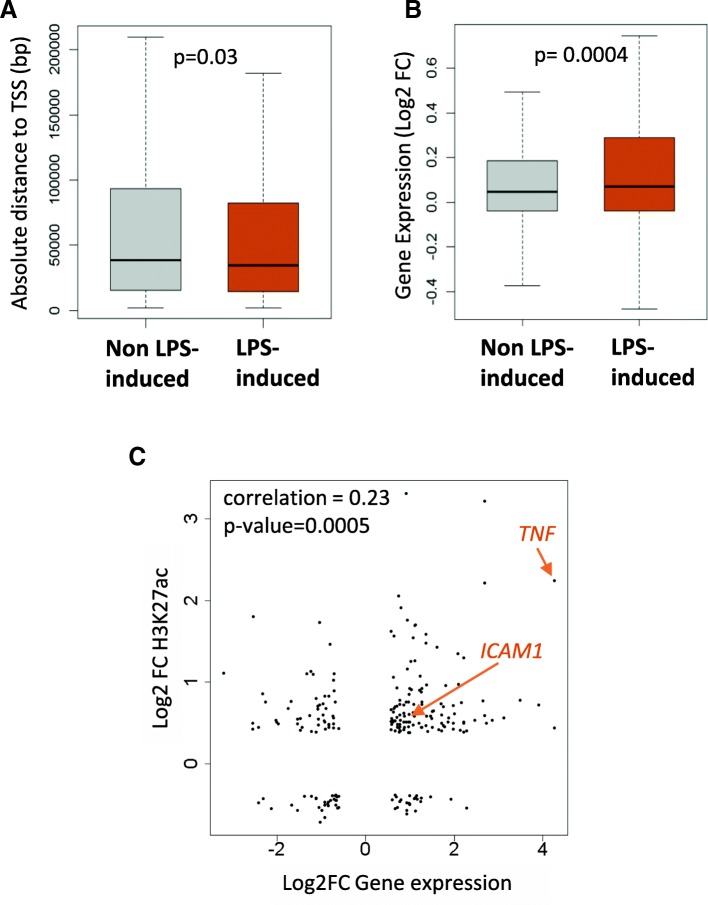
LPS-induced Hac and gene expression. **a**: Distance to the closest TSS. The distances were extracted for LPS-increased (orange) and non LPS-increased (grey) H3K27ac peaks. Only peaks outside of promoter regions (defined as -2Kb to +2Kb around the transcription Start Site) were considered. **b**: H3K27ac and gene expression. Each H3K27ac peak was assigned to the closest gene located within 1000Kb of the peak border. Expression of the genes associated with LPS-increased (orange) and non LPS-increased (grey) H3K27ac peaks was investigated. Difference between the two groups in A and B was tested with a Wilcoxon test for which the P-value is reported. **c**: Correlation between changes in H3K27ac gene expression. For each H3K27ac peak, the Log2 fold in expression of the genes assigned to it (x axis) was plotted against the Log2FC in H3K27ac ChIP-seq signal (y axis) after LPS stimulation. Correlation between these two data sets was tested with a Pearson test, the correlation coefficient together with the *P*-value is reported

Next, we examined the effect of the changes in H3K27ac on gene expression, using data from RNA-seq experiment generated previously [14]. First, we noticed that the LPS-increased enhancers identified were located closer to genes’ transcription start sites (TSS) as compared to non LPS-increased H3K27ac peaks (Fig. 2a and Additional file 6 A). Then, we assigned all H3K27ac peaks to the closest gene located within 1000Kb [18] and examine their expression. We found that the genes associated to LPS-increased regions were more likely to be over-expressed than the genes associated to non LPS-increased H3K27ac peaks (Fig. 2b and Additional file 2 D). Moreover, investigations revealed that H3K27ac was positively correlated with gene expression changes and although the correlation observed was weak, statistical significance was attained (Fig. 2c). Finally, differences in H3K27ac signal between LPS and Control conditions plotted along differentially expressed genes was seen only for up-regulated genes (Additional file 6 B), particularly close to the TSS and inside the gene. This further confirms that H3K27ac is an activation mark and shows the effect of stimulus-induced changes of this mark on stimulus-induced gene up-regulation.

One of the most striking changes in H3K27ac followed by gene up-regulation was found at the *TNF* locus described later on. However, other genes showing more modest LPS-increased H3K27ac and up-regulation could be observed. This was the case of the *ICAM1* gene showing an active region close to its promoter at rest for which the H3K27ac signal increased after LPS stimulation (Additional file 6 D). This effect was accompanied by a gene expression increase of about 2 fold (Additional file 6 C).

**Fig. 3:**
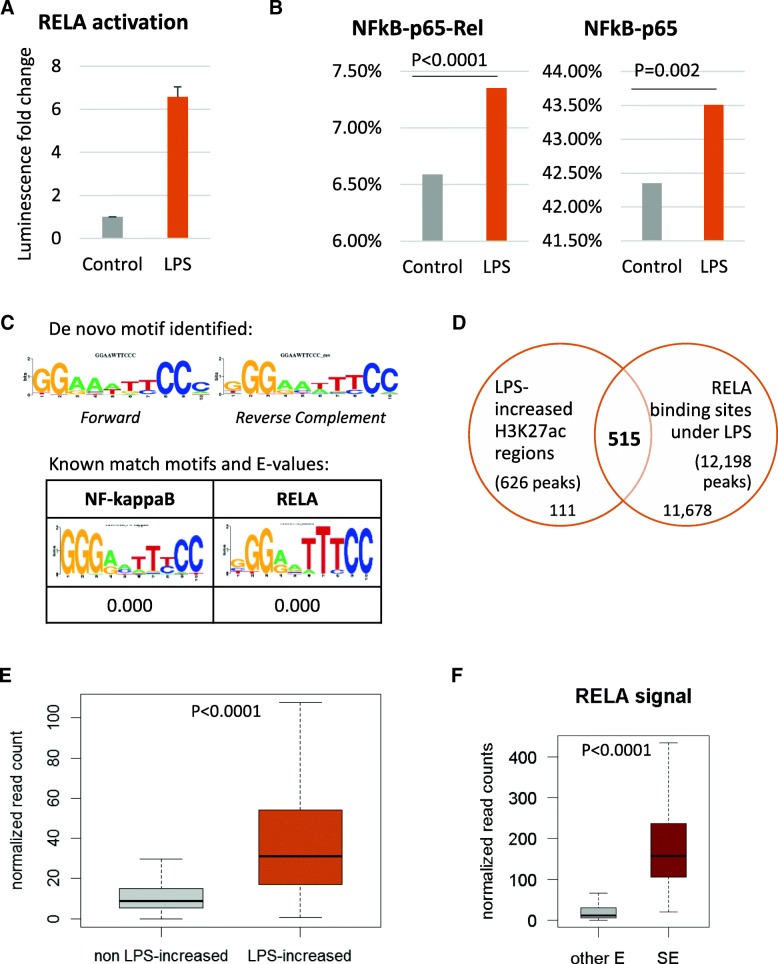
LPS-induced H3K27ac and RELA binding. **a**: RELA activation. Detroit 562 cells were treated with LPS at 1μg/ml for 80 min or not (Control). Nuclear proteins were extracted and RELA activation was tested. The bars show the average of 3 independent experiments as the fold change in luminescence after LPS stimulation compared to the control and the error bars represent the standard deviation. **b**: Motif analysis. Proportion of H3K27ac peaks identified under Control (grey) or LPS condition (orange) containing the known motifs NFKB-p65-Rel (left) or NFKB-p65 (right). Comparison between the two sets of peaks was performed using a Chi-square test and the P-values are reported. **c**: De novo motif analysis. The most enriched motif identified by de novo motif analysis in LPS-increased H3K27ac peaks against all H3K27ac peaks as background is reported (top) together with the known matched motifs and the E-value quantifying their resemblance (bottom). **d**: Overlap between H3K27ac and RELA peaks. LPS-increased H3K27ac regions and RELA binding sites identified under LPS stimulation were compared, two peaks were considered overlapping when they had at least one bp in common. **e**: RELA binding at H3K27ac peaks. RELA ChIP-seq data was used to quantify RELA binding at LPS-increased (orange) and non LPS-increased (grey) H3K27ac peaks. Difference between the two groups was tested with a Wilcoxon test. **f**: RELA binding at Super-Enhancers. RELA ChIP-seq data was used to quantify its binding at super-enhancers (SE – in red) and other enhancers (other E – in grey). Difference between the two groups was tested with a Wilcoxon test

We were then interested to investigate LPS-induced changes in Histone acetylation with regards to binding of the NF-kB member RELA, due to its important role in chromatin re-organization following stimulation [15, 16]. At the time point chosen for the H3K27ac ChIP-seq experiment, we could see a strong specific RELA activation (Fig. 3a and Additional file 7 A). Eighty minutes of LPS induction consisted of RELA first and highest activation peak (Additional file 7 B).

We first performed motif analysis on the H3K27ac peaks identified in Control and LPS conditions to examine any differences between the two. Interestingly, the top motifs identified were common and enriched similarly in both conditions and consisted mainly of the AP-1 family of transcription factors (Jun, Fosl2, Fra 1, ATF3, BATF, AP-1 – Additional file 8) also activated downstream of TLR stimulation [4]. In addition, we found the motif for RELA (also known as p65) subunit of NF-kB to show great variation between the enrichment in Control and LPS H3K27ac peaks (Additional file 8 – highlighted in yellow). The peaks identified under LPS stimulation were more likely to contain a motif for RELA compared to the control peaks (Fig. 3b). Moreover, the motif for p50/p52 – other NF-kB subunits – was also enriched in LPS but not in Control peaks (Additional file 8 – highlighted in green). Similarly, motifs analysis in the LPS-increased H3K27ac peaks revealed a great enrichment of RELA as well as p50/p52 motifs (Additional file 9). Furthermore, de novo motif analysis in the same set of peaks identified a consensus matching perfectly the NF-kB and RELA motifs (Fig. 3c). These observations suggest that NF-kB plays an important role in regulating gene expression following LPS detection, especially the RELA and p50/p52 subunits. This is consistent with current knowledge about the NF-kB canonical pathway involving RELA:p50 dimers that has been shown to be activated downstream of TLR4 following LPS binding [19].

We then used the RELA ChIP-seq data we generated previously [14] to investigate overlap between RELA binding and LPS-increased H3K27ac regions. Interestingly, 82.3% (515 out of 626) of the LPS-increased peaks overlapped with RELA binding sites in the same conditions (Fig. 3d). Moreover, RELA binding signal at the LPS-increased peaks was significantly higher than in other H3K27ac peaks (Fig. 3e). This was supported when plotting RELA signal along the H3K27ac peaks ordered according to their difference in signal between LPS and Control condition where the two data aligned (Additional file 4 C and quantification in Additional file 2 C). Furthermore, RELA signal at super-enhancers was significantly higher than at other enhancers (Fig. 3f) and it was also higher at LPS-only super-enhancers compared to Control-only super-enhancers (Additional file 7 C). Taken together these results support the role of RELA in coordinating the opening of the chromatin following stimulation [15, 16] as well as the formation of super-enhancers [20].

**Fig. 4:**
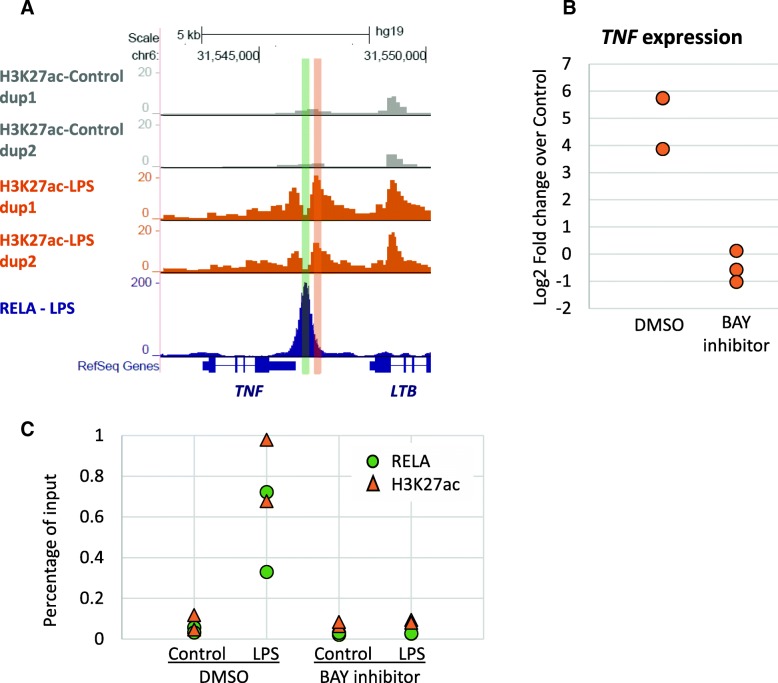
Example of the *TNF* locus. **a**: *TNF* locus. Example of an LPS-increased H3K27ac region as well as RELA binding just downstream of the *TNF* gene. **b**: *TNF* expression. Detroit 562 cells were pre-treated with BAY 11–7082 or DMSO before stimulation with LPS for 100 min, RNAs were extracted and RT-qPCR performed. Results show the log 2 fold change in gene expression over control in both conditions for 3 independent experiments. **c**: H3K27ac mark and RELA binding at the *TNF* locus. Detroit 562 cells were pre-treated with BAY 11–7082 or DMSO before stimulation with LPS for 80 min and ChIP-qPCR was performed for RELA (green – primers amplifying the green region in A) and H3K27ac (orange – primers amplifying the orange regions in **a**). Results show the percentage of input for the two ChIPs of two independent experiments

One of the most compelling examples of LPS-induced H3K27ac marks coinciding with RELA binding and increase gene expression was identified at the *TNF* locus. Indeed, we observed no H3K27ac present in the Control stimulation but a strong deposition of this mark after LPS treatment, co-localized with RELA binding (Fig. 4a). This was followed by a strong increase of *TNF* expression (Fig. 4b). Up-regulation of *TNF* seemed to reach its maximum at around 100 min of LPS stimulation (Additional file 10 A) which is 20 min after the time point investigated for H3K27ac ChIP-seq and where RELA is the most activated. Thus, we continued to look into the role of the NF-kB pathway in the deposition of this mark. For this purpose, we used a widely used NF-kB inhibitor, BAY 11–7082, to block the pathway. As expected, pre-treating the cells with the inhibitor before stimulating them with LPS abolished the recruitment of RELA at target genes such as *TNF* (Fig. 4c – green bars) and *NFKBIA* (Additional file 10 B). Additionally, up-regulation of these genes following LPS stimulation were also inhibited by the pre-treatment with BAY 11–7082 (Fig. 4b and Additional file 10 C). Presence of H3K27ac mark at the *TNF* locus in the same conditions was examined by ChIP-qPCR and revealed the same trend, with the deposition of this mark impaired when the cells were pre-treated with the NF-kB inhibitor before LPS stimulation (Fig. 4c). This finding suggests that RELA recruitment at the *TNF* locus is necessary for this epigenetic change to occur and for subsequent up-regulation of the *TNF* gene.

## Discussion

In this study, we identified LPS-induced H3K27ac sites in epithelial cells and reported 626 LPS-increased ones. These represent a small portion of the regulatory regions identified with this histone mark. They were highly enriched in enhancer regions, consistent with the important role of enhancers in cellular responses [5], and made up 1.7% of all activated enhancers. Several other studies have looked at enhancer activation following microbial or inflammatory stimuli, they reported various numbers of stimulus-induced activated enhancers but all consisted of a small fraction of all activated enhancers identified [7, 16, 21]. For instance, in the study by Ostuni et al., LPS induction was investigated in mouse macrophages and reported 6308 activated enhancers out of ~ 65,000 regulatory regions identified. They also reported 9004 enhancers that were repressed following LPS stimulation, contrasting with our study where we were not able to identify any LPS-decreased H3K27ac sites. Loss of activated enhancers following inflammatory stimuli has been documented and is usually associated with down-regulation of basal cell function [15, 20]. Differences in the number of stimulus-induced activated and repressed enhancers across studies and with our study may be due to the divergence of organisms and cell types used. Indeed, cell type specificity is known to influence enhancer selection [22] as well as gene expression response [23]. Alternatively, variation in the stimulus as well as the duration of induction could lead to differences in the chromatin remodeling observed between studies, especially considering that response to infection is a time-dependent process [24].

Furthermore, we compared the LPS-induced H3K27ac and RELA binding following the same stimulation. We found them to be highly correlated and most LPS-increased H3K27ac peaks to overlap that of RELA. Moreover, inhibiting the NF-kB pathway abolished the deposition of the histone mark following LPS stimulation, suggesting an important role for this transcription factor in regulating the above mentioned epigenetic change. These results are in line with that of another study looking at IL-1 signaling, a pleiotropic cytokine inducing inflammation through the NF-kB pathway. This study showed that RELA regulates IL-1 inducible H3K27ac, particularly via upstream members of the signaling pathway, TAK1 and IKK2 [16]. In addition, RELA is known to interact with chromatin modifying complexes such as p300 [25] resulting in acetylation of histones surrounding the sites where RELA binds DNA [26]. This could also explain the deposition of the H3K27ac surrounding RELA binding sites as seen in our results at the *TNF* locus and its impairment following NF-kB inhibition.

We also found RELA binding to be highly bound to Super-enhancers identified following LPS stimulation. This is consistent with previous reports showing that NF-kB is involved in chromatin rearrangement after TNFα induction [15], potentially by redistributing co-factors at gained super-enhancers [20].

Additionally, we found AP1 members motifs to be enriched in both Control and LPS H3K27ac peaks. The AP1 complex is activated downstream of TLR and is able to interact with NF-kB [27] but it also constitutively binds open chromatin, especially at intergenic and intronic regions [28] thus it is not surprising to find these motifs enriched in both conditions here.

Finally, we showed that H3K27ac deposition correlated with increased gene expression, consistent with the activating role of this mark [8]. Although the correlation coefficient between H3K27ac changes and gene expression regulation is rather limited, it is similar to what others investigating the same mark have reported [29]. Traditionally, regulation of inflammatory genes has been seen as a first wave of early genes that do not require chromatin remodeling expressed quickly after induction and a second wave of late genes showing slower kinetics due to changes in the chromatin that are necessary to make them accessible [26]. However, in our study, *TNF* – which is considered an early gene in response to LPS stimulation [30] – did show H3K27ac changes close to its promoter which was not observed at resting condition suggesting that some degree of chromatin rearrangement was necessary to induce gene expression in this case. This is further confirmed by the suppression of its up-regulation following LPS when H3K27ac deposition is abolished following NF-kB inhibition. The rapidity of TNF expression following LPS stimulation could indicate that the promoter was already in a poised state where other histone marks were present [31].

## Conclusion

We identified promoters and enhancers activated following sensing of LPS, a gram negative bacterial endotoxin, via TLR4. We found that deposition of H3K27ac marks correlated with increased gene expression as well as recruitment of RELA, one of the NF-kB members particularly important for the innate immune response. In addition to validate previous studies showing epigenetic changes following external signals, this data set may be valuable in the context of respiratory infectious diseases.

## Methods

### Cell culture and treatment

Detroit 562 cells were purchased from ATCC and cultured in RPMI medium (Gibco) supplemented with 10% fetal bovine serum performance (Gibco), 100 U/ml penicillin and 100μg/ml streptomycin (Gibco), and 1 mM sodium pyruvate (Gibco). LPS from E.coli B4:111 (Sigma) at 1 μg/mL was used to stimulate the cells. Inhibition of NF-kB was done treating the cells with BAY 11–7082 (ChemCruz) at 100uM (stock solution at 50 mM in DMSO) for one hour prior and at 90uM (dilution 9/10 in the medium containing the ligand) during the LPS treatment (Fig. 4 b and c). RELA activation assay, RT-qPCR and ChIP-qPCR procedures are described in Additional file 11.

### ChIP-seq

Cells were treated with LPS or with fresh medium (Control - no treatment) for 80 min in Detroit 562 cells. ChIP-seq was performed according to the library-on-beads protocol from Wallerman et al. [32] using the NEBnext DNA library prep kit (New England Biolabs) as described previously [14] with the following modifications. 5 μg of H3K27ac antibody (Active motif, reference 39,138) was mixed with 50 uL of Dynabeads Protein G before immunoprecipitation. Sequencing was performed on an Illumina NextSeq sequencer (1x76bp). Biological duplicates consisting of two independent experiments were sequenced and analyzed.

Sequencing reads were mapped to the human genome hg19 with BWA, quality control and filtering were then performed using samtools. Multi-aligned or unmaped reads, PCR duplicates and reads with a MAPQ< 20 were filtered out giving around 50–110 millions passed reads per sample (Additional file 1). For each sample, peak calling was performed with Dfilter [33] against input DNA using relaxed threshold (−lpval = 1). The irreproducibility discovery rate (IDR) method [34] was used to identify reproducible peaks in both conditions separately. Peaks with an IDR < 0.05 between duplicates were selected in Control and LPS and consisted of the final peaks in each condition. Any peaks overlapping the blacklisted regions found in wgEncodeDacMapabilityConsensusExcludable.bed (http://hgdownload.cse.ucsc.edu/goldenpath/hg19/encodeDCC/wgEncodeMapability/) were further removed from the final list (Additional file 1).

Homer [35] was used to generate bedGraph files to visualize the signal in the UCSC genome browser with the command makeTagdirectory followed by makeUCSCfile (Fig. 4a and Additional file 10 C). In the same program, the command mergePeaks.pl allowed the comparison of different sets of peaks (Figs. 1a, e and 3d). The output merged peaks of the comparison between Control and LPS H3K27ac peaks consisted of the common as well as condition-specific peaks and were referred to as “all H3K27ac peaks”. The command annotatePeaks.pl in Homer was used to annotate the peaks (Fig. 1d, Additional file 4 B and Additional file 2 B).

Differential binding analysis was done on all H3K27ac peaks using each duplicate’s bam files from LPS and Control H3K27ac ChIP-seq as well as corresponding input DNA bam files as background. GC and read count normalization was first performed, then for every one of H3K27ac peaks (row), tag count for each condition was normalized by the median of the row, and the fold change over the median for each condition was generated and used in the heatmap representation. A threshold of 1.5 (signal of more than 1.5 time the median) was applied and clustering was then performed with K-mean clustering, dividing the data into 4 groups (Fig. 1b).

Throughout the manuscript, LPS-increased H3K27ac peaks refer to reproducible peaks showing an increase in signal in the LPS compared to the Control H3K27ac ChIP-seq in the differential binding analysis (Group 1 in Fig. 1b). Non LPS-increased H3K27ac peaks refer to all other H3K27ac peaks not present in this group of peaks.

Gene Ontology was carried out with GREAT, genes were associated to the peaks using the “single nearest gene” rule with a threshold of 1000Kb and all H3K27ac (peaks identified in LPS and/or Control ChIP-seq) were used as background (Fig. 1c and Additional file 3).

Motif analysis was performed with Homer using findMotifsGenome.pl command. Known motif enrichment in the LPS and Control H3K27ac peaks was done against the whole genome (Fig. 3b and Additional file 7) while known and de novo motif enrichment in the LPS-regulated H3K27ac peaks was compared to all H3K27ac peaks (Fig. 3c and Additional file 8).

Super enhancers analysis was performed as described previously [36]. All H3K27ac peaks identified in LPS and/or Control condition were used as input and the H3K27ac ChIP-seq signal (merged bam file from duplicates) used to rank the enhancers (Additional file 5).

### Integration of ChIP-seq with gene expression data

The Gene expression data used in this study consists of RNA-seq experiments performed in control and LPS (treatment for 100 min with 1μg/mL) condition in the same Detroit 562 cells [14] and available under the accession number GSE91019 in the NCBI’s Gene Expression Omnibus (GEO) depository.

Distance to the closest TSS was retrieved from the annotation analysis performed with the command annotatePeaks.pl in Homer. Only the peaks not located at a gene’s promoter (-2Kb to +2Kb around TSS) are considered when comparing LPS-increased and non LPS-increased H3K27ac peaks in Fig. 3a while the whole distribution can be found in Additional file 9 A.

The Rnachipintegrator program (https://github.com/fls-bioinformatics-core/RnaChipIntegrator) was used to assign H3K27ac peaks to their closest gene within 1000 Kb of the peak edge. Log2FC or FPKM values for the genes associated were then extracted from the RNA-seq data in order to investigate their expression (Fig. 3b and Additional file 4 D and Additional file 2 D). Log2FC was also used to interrogate the correlation between H3K27ac signal and gene expression. In this analysis, log2FC H3K27ac was calculated from the normalized read count in each condition (see Additional file 11 for details). Moreover, the following thresholds were applied to remove noise: Log2FC H3K27ac < 0.38 (FC = 1.3) and Log2FC Gene expression < 0.58 (FC = 1.5).

Up- down- and non-regulated genes from the RNA-seq data were also used to investigate their H3K27ac profile in both condition (Additional file 9 B) with ngs.plot program [37].

### Integration of H3K27ac ChIP-seq with RELA binding

The RELA DNA binding data used in this study is available under the accession number GSE91018 in the NCBI’s GEO repository. It consists of RELA ChIP-seq experiments performed under Control or LPS (treatment for 80 min at 1μg/mL) condition in the same Detroit 562 cells [14].

RELA ChIP-seq was used to quantify RELA binding at the various H3K27ac peaks identified in this study. Normalized read count in this data-set was obtained with the command annotatePeaks.pl from Homer, using the option –d referring to the duplicates’ merged RELA ChIP-seq data set’s tag directory. Read counts were used to compare RELA binding at LPS-increased and non LPS-increased H3K27ac regions (Fig. 3e) and at super-enhancers and other enhancers (Fig. 3f) as well as to quantify the signals in the integrated analysis (see Additional file 11 for details).

### Statistical analysis

Comparison of proportions in Figs. 1d and 3b was tested using the online 2 × 2 Chi-square test from Vassarstats (http://vassarstats.net/odds2x2.html) and the Pearson *P*-value was reported. Significance between distributions (Figs. 2a, b, 3e and f) was investigated using a non –paired Wilcoxon rank-sum test (wilcox.test in R). Finally, correlation between H3K27ac signal and gene expression was tested with a Pearson test (cor.test in R), correlation coefficient as well as P-value is reported (Fig. 2c).

## Additional files

Additional file 1: H3K27ac ChIP-seq statistics. Summary of the number of reads and peaks from the H3K27ac ChIP-seq experiments performed together with input DNA controls. (XLSX 8 kb)

Additional file 2: Summary of the changes observed – Quantification. The same sets of increased, unchanged and decreased H3K27ac peaks as in Additional file 2 were analyzed. Data shown in the latter figures were quantified in each group of peaks. A: H3K27ac changes after LPS treatment. Absolute value of the log2 (ratio LPS/Control normalized read counts) were used to draw the box plots of the H3K27ac changes in the three sets of peaks. B: Annotation of the peaks. Number of peaks identified as Promoters (orange), Enhancers (yellow) and Others (green) were plotted on the histogram for the three sets of peaks. C: NFkB-RELA signal. The median of normalized RELA ChIP-seq read counts inside each peak is represented with the box plot for the three groups. D: Gene expression. FPKM values for each gene associated with the H3K27ac peaks were extracted in both Control and LPS conditions and were used to draw the box plot for each of the three sets of peaks. See Additional file 11 for more details on the integration analysis. (PDF 358 kb)

Additional file 3: Gene Ontology analysis on LPS-increase H3K27ac peaks. The 626 LPS-increased H3K27ac peaks identified in the differential binding analysis (Fig. 1b) were used in GREAT and associated with the nearest gene within 1000Kb. Significant gene ontology terms for biological processes output are shown. (XLSX 31 kb)

Additional file 4: Summary of the changes observed. A: H3K27ac changes after LPS treatment. Peaks were ranked according to the ratio LPS/Control of the H3K27ac ChIP-seq normalized read counts and top (Increased), middle (Unchanged) and bottom (Decreased) 300 peaks were extracted. Log2 Fold change (Log2FC) of the H3K27ac change is represented with the histogram (see Additional file 12 for the peaks information). B: Annotation of the peaks. The peaks in A were annotated and represented on the graph by color-code as follow: enhancers if they were intergenic, in introns, 3’ UTR or TTS (yellow), promoters if identified in promoters or 5’ UTR of a gene (orange) or others (green). C: NFkB-RELA signal. Normalized RELA ChIP-seq read counts inside each peak were used as a measure of RELA binding. The peaks in A were binned into groups of 25 and average of read count for each group was plotted on the graph. D: Gene expression. Each of the peaks in A were assigned to the closest gene and the log2 fold change of gene expression for this was plotted. Genes for which FPKM in one condition (LPS or Control) was 0 were removed and are shown as gaps. See Additional file 11 for more details on the integration analysis. (PDF 423 kb)

Additional file 5: Super-Enhancers analysis. A: Ranked plot of enhancers defined in Control (left) and LPS (right) condition according to their H3K27ac ChIP-seq signal. B: Distribution of the length of the super-enhancers (*n* = 1119) and non super-enhancers (other E, *n* = 17,781) identified in LPS condition. C: Top 10 biological processes terms from Gene Ontology analysis of super-enhancers identified in Control (left) or LPS (right) condition. D: Example of a super-enhancer only called under LPS but not in Control. The region highlighted in the dashed box upstream of *CSF2* gene shows increased H3K27ac signal after LPS stimulation and was therefore called as a Super-Enhancer. (PDF 435 kb)

Additional file 6: LPS-induced H3K27ac and gene expression. A: Distribution of the fraction of the LPS-induced (orange) or all (grey) H3K27ac peaks around the TSS of the closest gene. All the peaks were included in the top panel while only the promoter peaks (located between − 2000 to + 2000 bp of the TSS) were removed in the bottom panel. B: H3K27ac ChIP-seq signal under LPS (L) or no treatment (C) condition was plotted 50Kb upstream to 50Kb downstream of a gene according to the gene status: up-regulated (UP-red, 239 genes), non-regulated (NO-grey, 62,646 genes) or down-regulated (DOWN-green, 206 genes) after LPS stimulation. B: ICAM1 gene expression. Detroit 562 cells were treated with LPS at 1μg/ml for 2 h, RNA were extracted and RT-qPCR performed. Results show fold change in gene expression over the no treatment (Control) condition, normalized with housekeeping gene, for 3 independent experiments. C: ICAM1 locus. Example of LPS-increased H3K27ac regions upstream as well as at the promoter of ICAM1 gene. (PDF 629 kb)

Additional file 7: RELA activation following LPS treatment and binding at super-enhancers. A: Detroit 562 cells were treated with LPS at 1 μg/mL or fresh medium (no treatment) for 2 h and nuclear protein were extracted to be used in NFkB p65 transcription factor assay. No binding sites (no BS), wild type NFkB binding sites (WT BS) or mutant NFkB binding sites (mutant BS) were added in solution into the wells to check for RELA specificity. B: The cells were treated similarly and RELA activation was investigated at different time points for an extended period of time. The two curves correspond to independent experiments (dup = duplicate). C: Distribution of the RELA signal in Control-only (grey) and LPS-only (orange) Super-enhancers identified from the H3K27ac ChIP-seq data. (PDF 318 kb)

Additional file 8: Motifs analysis on Control and LPS H3K27ac peaks. Motif enrichment analysis for known transcription factor motifs was performed on the H3K27ac peaks identified in LPS and Control condition against the whole genome as background. -log 10 (*P*-value) of the enrichment is reported in both condition for each motif. NF-kB p65 and NF-kB p50/p52 motifs are highlighted in yellow and green respectively. (XLSX 13 kb)

Additional file 9: Motifs analysis on LPS-increased H3K27ac peaks. Motif enrichment analysis for known (left) as well as de novo (right) transcription factor motifs was performed on the 626 LPS-increased H3K27ac peaks against all H3K27ac peaks as background. Enrichment for each motif is reported, NF-kB p65 and NF-kB p50/p52 motifs are highlighted in yellow and green respectively. (XLSX 13 kb)

Additional file 10: Target genes expression and RELA occupancy. A: Time course of TNF expression. Detroit 562 cells were treated with LPS for different time points where RNA were extracted and RT-qPCR performed. The curve shows the variation in Fold change over the expression of TNF at rest over time, for one experiment. B: RELA binding at the *NFKBIA* locus. Detroit 562 cells were pre-treated with BAY 11–7082 or DMSO before stimulation with LPS for 80 min and ChIP-qPCR was performed for RELA. Results show the average percentage of input of two independent experiments, error bars are standard deviation. C: *NFKBIA* expression. Detroit 562 cells were pre-treated with BAY 11–7082 or DMSO before stimulation with LPS for 100 min, RNAs were extracted and RT-qPCR performed. Results show the average fold change in gene expression over control in both conditions and for 3 independent experiments. Error bars represent standard deviation. (PDF 387 kb)

Additional file 11: Supplementary methods. Details of the procedures for RELA activation assay, RT-qPCR, ChIP-qPCR as well as the integration analyses performed to generate Additional files 2 and 4. (PDF 458 kb)

Additional file 12: H3K27ac peaks summary. Summary of the H3K27ac peaks identified: coordinates, Annotation, changes in H3K27ac as log2FC of the ration of normalized count between LPS and Control condition, RELA signal, gene associated to each peak and its expression as log2FC after LPS stimulation compared to Control and FPKM in LPS and Control conditions. (XLSX 3301 kb)

## Abbreviations

H3K27ac: Histone 3 Lysine 27 acetylation
Log2FC: Log 2 (Fold change)
LPS: Lipopolysaccharide
NF-kB: Nuclear Factor kappa B
TLR: Toll-like Receptor
TSS: Transcription Start Site
