# Supplementary figures and images for: Changes in H3K27ac following lipopolysaccharide stimulation of nasopharyngeal epithelial cells
Source: BMC Genomics. 2018 Dec 27;19:969. doi: 10.1186/s12864-018-5295-4 (PMC6307289; doi:10.1186/s12864-018-5295-4)

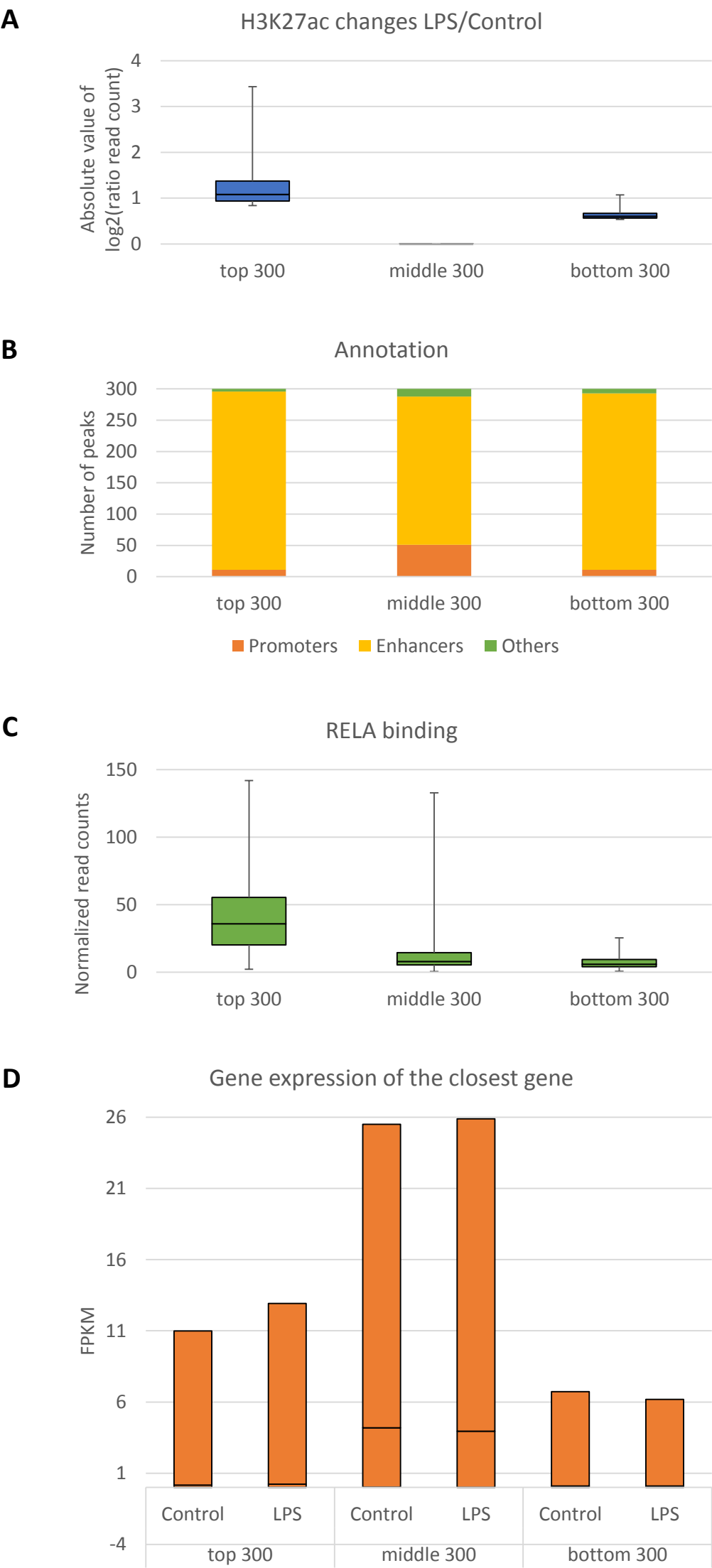

Supplement: Supplementary file 2 — Summary of the changes observed – Quantification. The same sets of increased, unchanged and decreased H3K27ac peaks as in Additional file 2 were analyzed. Data shown in the latter figures were quantified in each group of peaks. A: H3K27ac changes after LPS treatment. Absolute value of the log2 (ratio LPS/Control normalized read counts) were used to draw the box plots of the H3K27ac changes in the three sets of peaks. B: Annotation of the peaks. Number of peaks identified as Promoters (orange), Enhancers (yellow) and Others (green) were plotted on the histogram for the three sets of peaks. C: NFkB-RELA signal. The median of normalized RELA ChIP-seq read counts inside each peak is represented with the box plot for the three groups. D: Gene expression. FPKM values for each gene associated with the H3K27ac peaks were extracted in both Control and LPS conditions and were used to draw the box plot for each of the three sets of peaks. See Additional file 11 for more details on the integration analysis. (PDF 358 kb) [file 12864_2018_5295_MOESM2_ESM.pdf]

Additional File 4

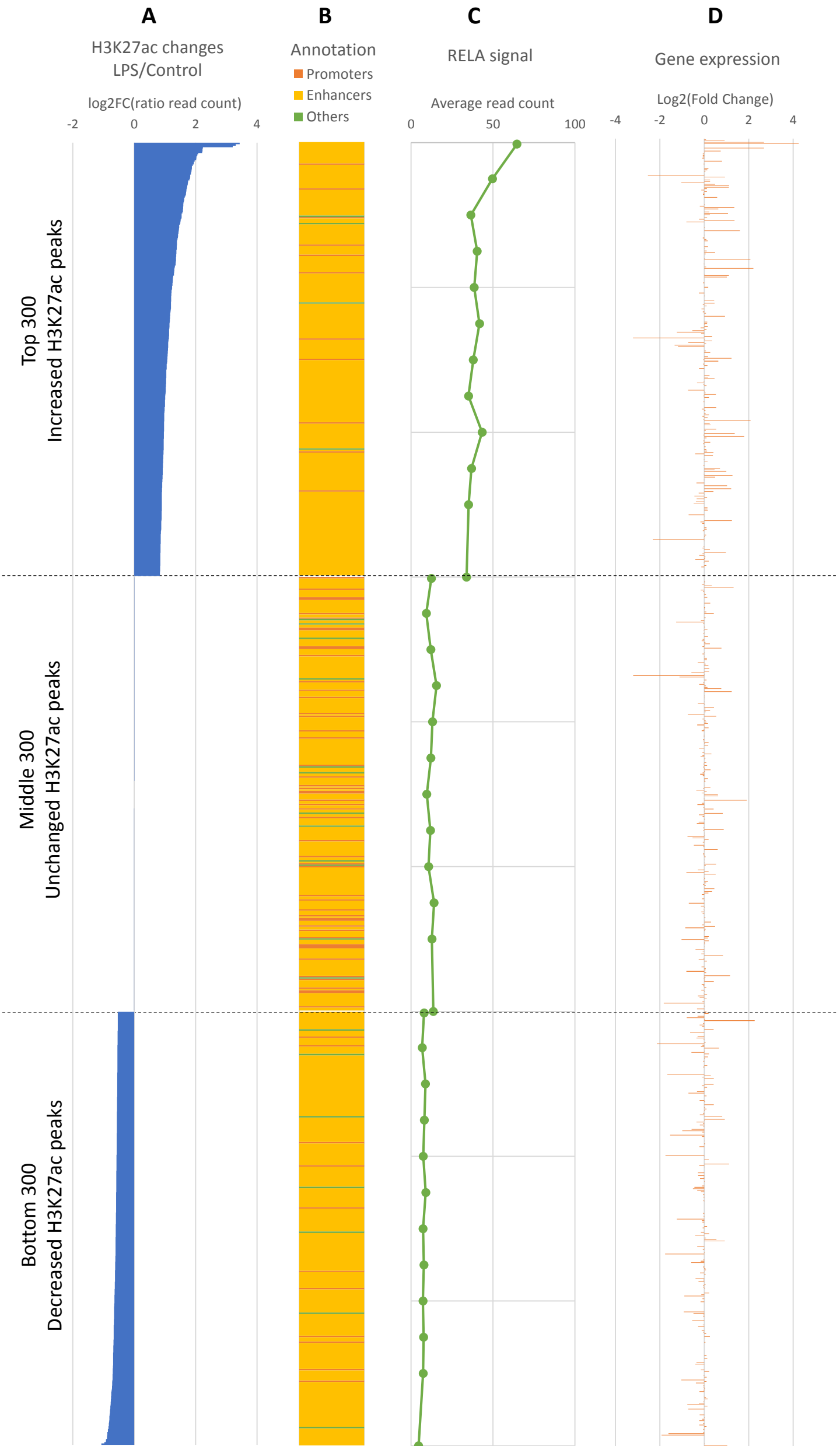

Supplement: Supplementary file 4 — Summary of the changes observed. A: H3K27ac changes after LPS treatment. Peaks were ranked according to the ratio LPS/Control of the H3K27ac ChIP-seq normalized read counts and top (Increased), middle (Unchanged) and bottom (Decreased) 300 peaks were extracted. Log2 Fold change (Log2FC) of the H3K27ac change is represented with the histogram (see Additional file 12 for the peaks information). B: Annotation of the peaks. The peaks in A were annotated and represented on the graph by color-code as follow: enhancers if they were intergenic, in introns, 3’ UTR or TTS (yellow), promoters if identified in promoters or 5’ UTR of a gene (orange) or others (green). C: NFkB-RELA signal. Normalized RELA ChIP-seq read counts inside each peak were used as a measure of RELA binding. The peaks in A were binned into groups of 25 and average of read count for each group was plotted on the graph. D: Gene expression. Each of the peaks in A were assigned to the closest gene and the log2 fold change of gene expression for this was plotted. Genes for which FPKM in one condition (LPS or Control) was 0 were removed and are shown as gaps. See Additional file 11 for more details on the integration analysis. (PDF 423 kb) [file 12864_2018_5295_MOESM4_ESM.pdf]

Additional File 6

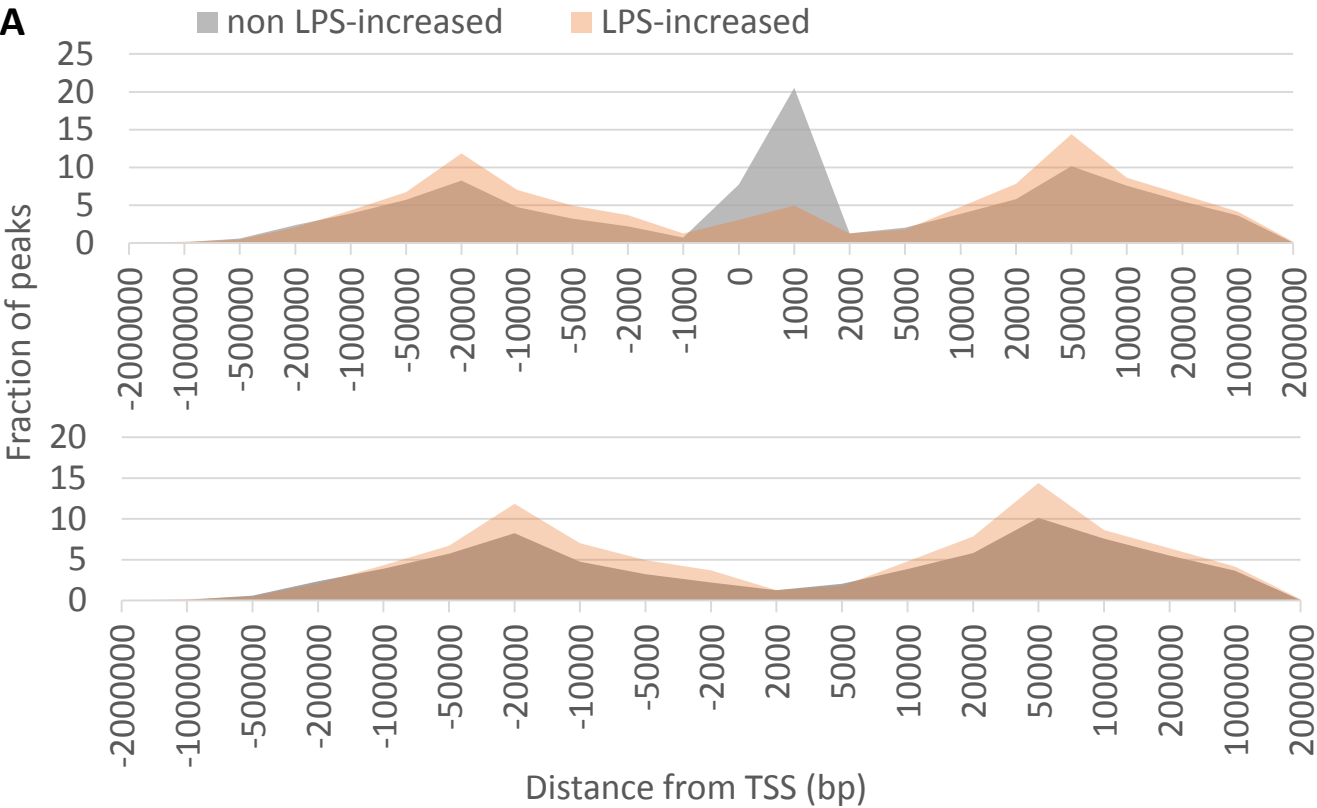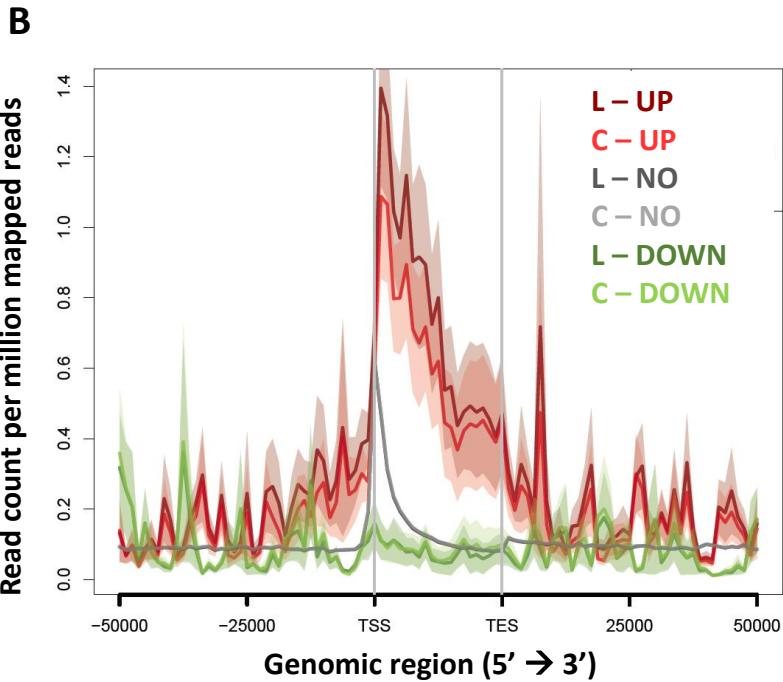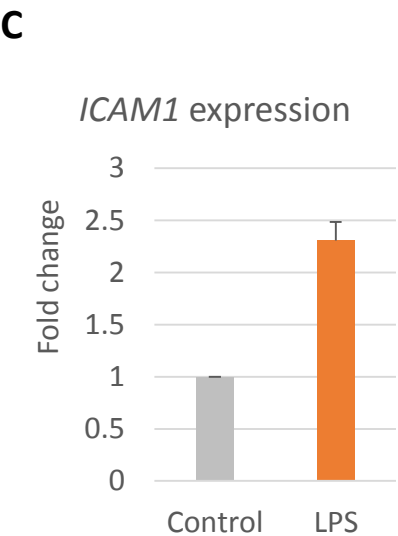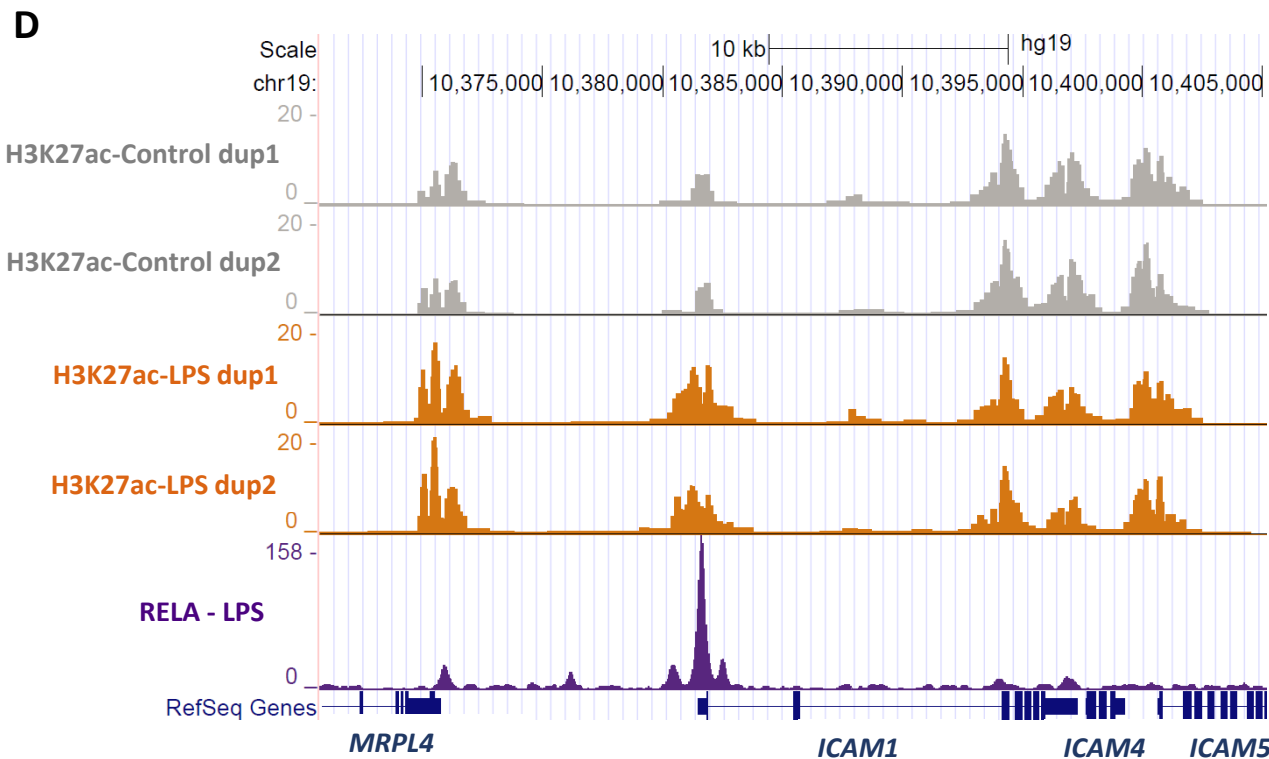

Supplement: Supplementary file 6 — LPS-induced H3K27ac and gene expression. A: Distribution of the fraction of the LPS-induced (orange) or all (grey) H3K27ac peaks around the TSS of the closest gene. All the peaks were included in the top panel while only the promoter peaks (located between − 2000 to + 2000 bp of the TSS) were removed in the bottom panel. B: H3K27ac ChIP-seq signal under LPS (L) or no treatment (C) condition was plotted 50Kb upstream to 50Kb downstream of a gene according to the gene status: up-regulated (UP-red, 239 genes), non-regulated (NO-grey, 62,646 genes) or down-regulated (DOWN-green, 206 genes) after LPS stimulation. B: ICAM1 gene expression. Detroit 562 cells were treated with LPS at 1μg/ml for 2 h, RNA were extracted and RT-qPCR performed. Results show fold change in gene expression over the no treatment (Control) condition, normalized with housekeeping gene, for 3 independent experiments. C: ICAM1 locus. Example of LPS-increased H3K27ac regions upstream as well as at the promoter of ICAM1 gene. (PDF 629 kb) [file 12864_2018_5295_MOESM6_ESM.pdf]

A

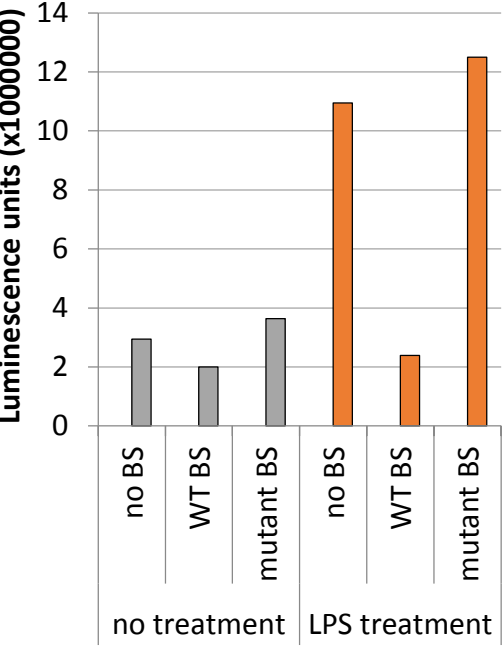

B

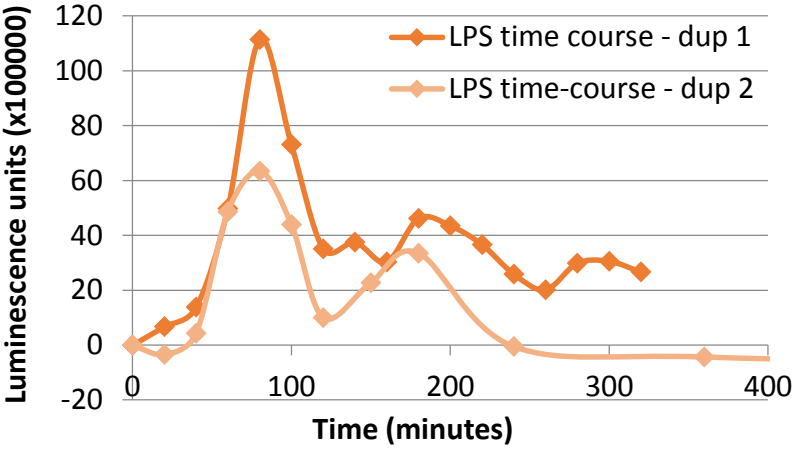

C

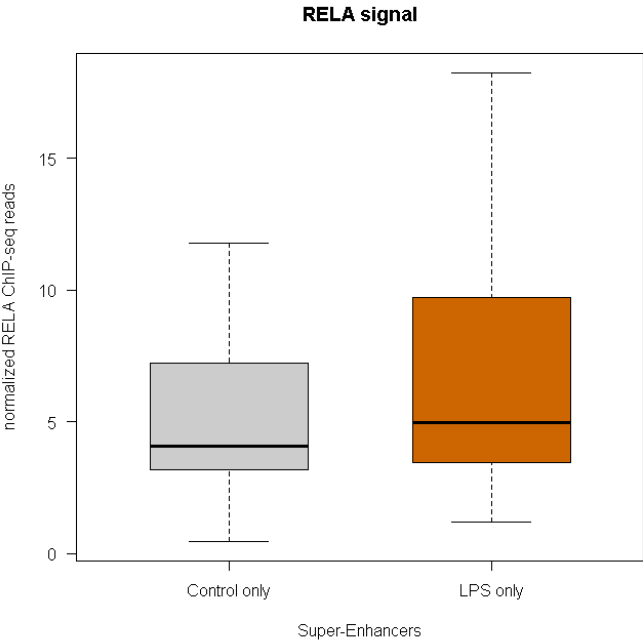

Supplement: Supplementary file 7 — RELA activation following LPS treatment and binding at super-enhancers. A: Detroit 562 cells were treated with LPS at 1 μg/mL or fresh medium (no treatment) for 2 h and nuclear protein were extracted to be used in NFkB p65 transcription factor assay. No binding sites (no BS), wild type NFkB binding sites (WT BS) or mutant NFkB binding sites (mutant BS) were added in solution into the wells to check for RELA specificity. B: The cells were treated similarly and RELA activation was investigated at different time points for an extended period of time. The two curves correspond to independent experiments (dup = duplicate). C: Distribution of the RELA signal in Control-only (grey) and LPS-only (orange) Super-enhancers identified from the H3K27ac ChIP-seq data. (PDF 318 kb) [file 12864_2018_5295_MOESM7_ESM.pdf]
