# Supplementary material for: Changes in H3K27ac following lipopolysaccharide stimulation of nasopharyngeal epithelial cells
Source: BMC Genomics. 2018 Dec 27;19:969. doi: 10.1186/s12864-018-5295-4 (PMC6307289; doi:10.1186/s12864-018-5295-4)

Additional file 5

A

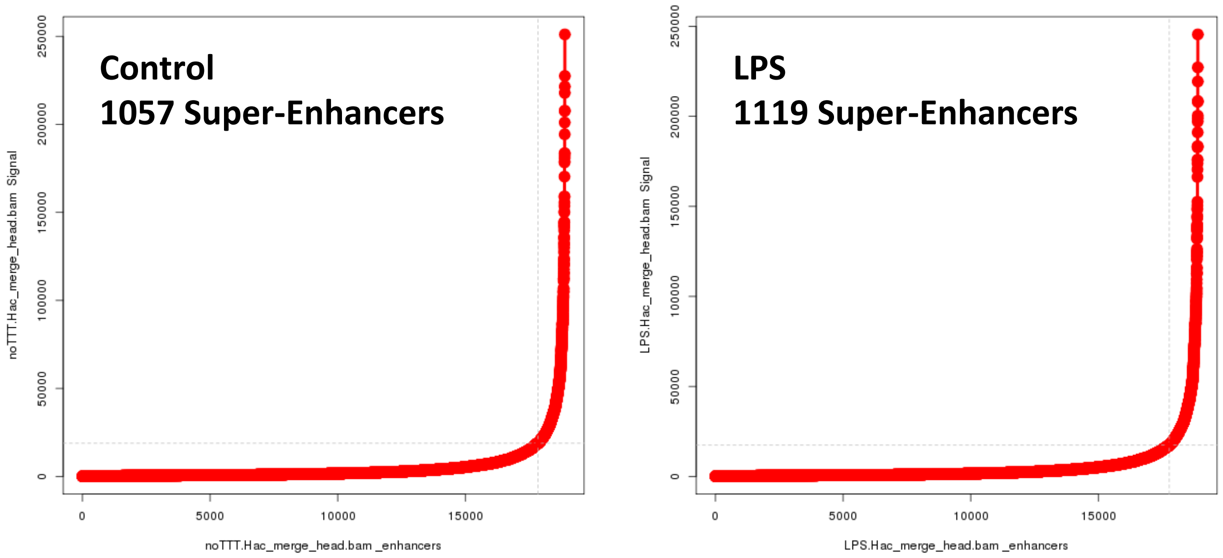

B

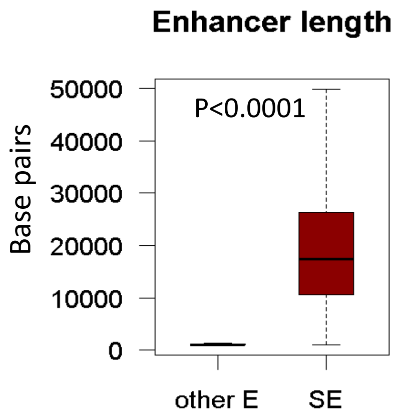

C

GO analysis on SE identified in Control	
# Term Name	Binom Raw P-Value
regulation of phosphorylation	2.55443e-15
negative regulation of response to stimulus	1.70549e-14
regulation of response to stress	1.77524e-14
regulation of cell migration	2.02591e-14
regulation of cell motility	6.28205e-14
tissue morphogenesis	1.10181e-13
positive regulation of cellular component movement	1.18620e-13
regulation of intracellular protein kinase cascade	2.90763e-13
negative regulation of signal transduction	3.13608e-13
negative regulation of apoptotic process	3.72527e-13

GO analysis on SE identified in LPS	
# Term Name	Binom Raw P-Value
immune system process	2.69856e-22
regulation of apoptotic process	1.74706e-18
regulation of programmed cell death	2.29836e-18
regulation of phosphorylation	1.31696e-17
regulation of cell migration	1.47684e-17
regulation of cell death	2.19674e-17
regulation of cell motility	2.23163e-17
regulation of response to stress	1.03661e-16
positive regulation of cellular component movement	1.54863e-16
tissue morphogenesis	2.42333e-16

D

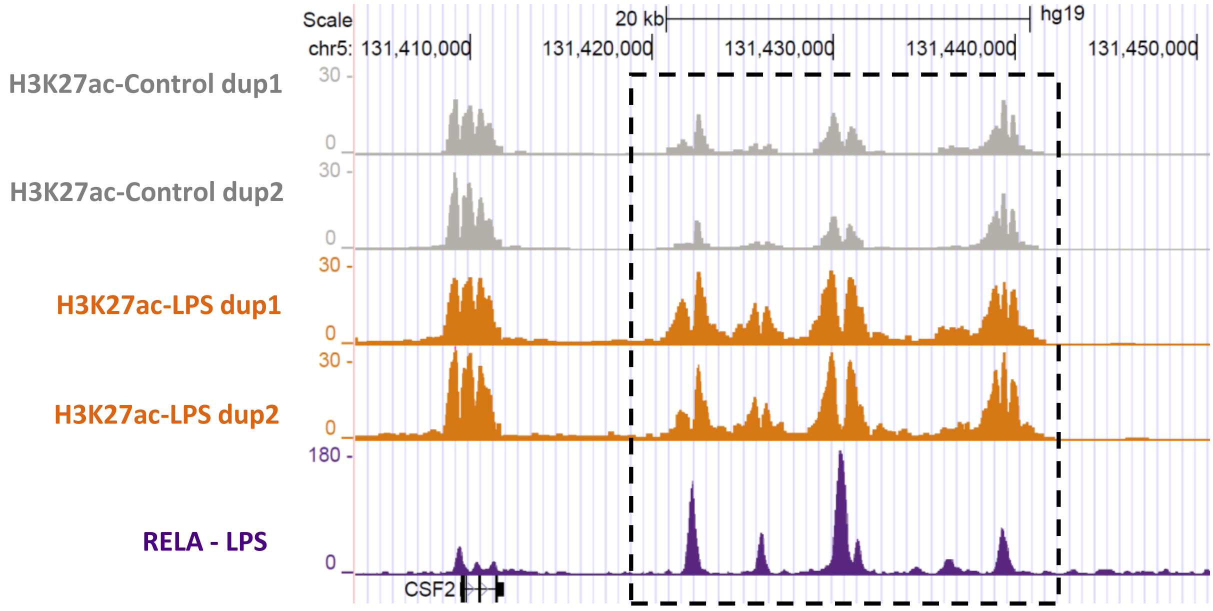

Supplement: Supplementary file 5 — Super-Enhancers analysis. A: Ranked plot of enhancers defined in Control (left) and LPS (right) condition according to their H3K27ac ChIP-seq signal. B: Distribution of the length of the super-enhancers (n = 1119) and non super-enhancers (other E, n = 17,781) identified in LPS condition. C: Top 10 biological processes terms from Gene Ontology analysis of super-enhancers identified in Control (left) or LPS (right) condition. D: Example of a super-enhancer only called under LPS but not in Control. The region highlighted in the dashed box upstream of CSF2 gene shows increased H3K27ac signal after LPS stimulation and was therefore called as a Super-Enhancer. (PDF 435 kb) [file 12864_2018_5295_MOESM5_ESM.pdf]
