# Supplementary material for: Changes in H3K27ac following lipopolysaccharide stimulation of nasopharyngeal epithelial cells
Source: BMC Genomics. 2018 Dec 27;19:969. doi: 10.1186/s12864-018-5295-4 (PMC6307289; doi:10.1186/s12864-018-5295-4)

Additional file 10

A

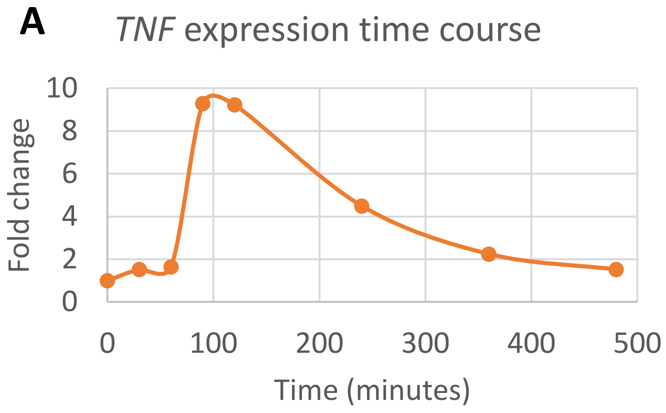

B

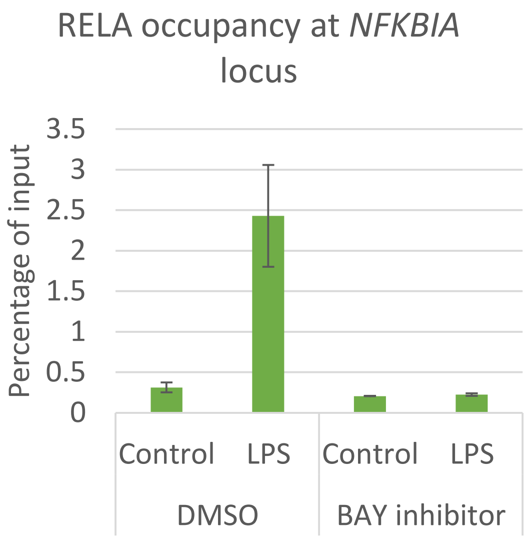

C

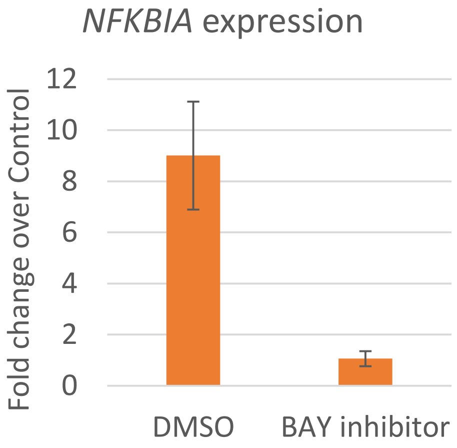

Supplement: Supplementary file 10 — Target genes expression and RELA occupancy. A: Time course of TNF expression. Detroit 562 cells were treated with LPS for different time points where RNA were extracted and RT-qPCR performed. The curve shows the variation in Fold change over the expression of TNF at rest over time, for one experiment. B: RELA binding at the NFKBIA locus. Detroit 562 cells were pre-treated with BAY 11–7082 or DMSO before stimulation with LPS for 80 min and ChIP-qPCR was performed for RELA. Results show the average percentage of input of two independent experiments, error bars are standard deviation. C: NFKBIA expression. Detroit 562 cells were pre-treated with BAY 11–7082 or DMSO before stimulation with LPS for 100 min, RNAs were extracted and RT-qPCR performed. Results show the average fold change in gene expression over control in both conditions and for 3 independent experiments. Error bars represent standard deviation. (PDF 387 kb) [file 12864_2018_5295_MOESM10_ESM.pdf]
