# Supplementary material for: Changes in H3K27ac following lipopolysaccharide stimulation of nasopharyngeal epithelial cells
Source: BMC Genomics. 2018 Dec 27;19:969. doi: 10.1186/s12864-018-5295-4 (PMC6307289; doi:10.1186/s12864-018-5295-4)
Supplement: Supplementary file 11 — Supplementary methods. Details of the procedures for RELA activation assay, RT-qPCR, ChIP-qPCR as well as the integration analyses performed to generate Additional files 2 and 4. (PDF 458 kb) [file 12864_2018_5295_MOESM11_ESM.pdf]

Changes in H3K27ac following Lipopolysaccharide Stimulation of Nasopharyngeal Epithelial Cells

Lisa Borghini et al.

Additional file 11: Supplementary methods

RELA activation assay

Cells were treated with LPS and nuclear proteins were extracted using the NE-PER kit (Pierce) and quantified with the Coomasie Protein quantification kit (Pierce). Ten to 15ug of proteins were used to test RELA activation using the NFκB p65 transcription factor assay kit (Pierce) according to the manufacturer's instructions (Figure 3A and Additional File 7 A and B).

Integrated analysis in Additional files 2 and 4

Read counts for all H3K27ac peaks were obtained from the annotatePeaks.pl command in Homer using the option –d referring to the merged H3K27ac ChIP-seq data file for both LPS and Control. Using these, the log2 fold change (log2FC) in signal after LPS treatment compared to Control was calculated as follow: log2(read count in LPS/read count in Control) (Additional file 12). It was used to rank the peaks according to their change in H3K27ac signal and was plotted for the 300 top, middle and bottom peaks in Additional file 4 A. In addition, the same measure was used to generate the boxplot of absolute log2FC in H3K27ac for the same peaks set in Additional file 2 A.

Annotation of the peaks was extracted from the output generated with the annotatePeaks.pl command. For visualization purposes, annotations were simplified to Enhancers (intronic, intergenic and TTS and 3'-UTR peaks), Promoters (promoters-TSS and 5'-UTR peaks) and Others (other peaks). Annotations for the top, middle and bottom 300 peaks based of their H3K27ac signal change after LPS were plotted in Additional file 4 B along the peaks and in Additional file 2 B to show the variation in proportion between the different sets of peaks.

RELA ChIP-seq was used to quantify RELA binding at all H3K27ac peaks. Normalized read count in this data-set was obtained with the command annotatePeaks.pl from Homer, using the option –d referring to the duplicates' merged RELA ChIP-seq data set's tag directory. Read counts were plotted along the top, middle and bottom 300 peaks based on their H3K27ac signal change after

LPS. For this analysis, peaks were binned in groups of 25 and the average RELA ChIP-seq read count was computed for each group and plotted in Additional file 4 C. The read counts were also used to generate the boxplot in Additional file 2 C.

Log2FC and FPKM values for gene expression were extracted from the RNA-seq data performed previously and assigned to the genes associated to each H3K27ac peaks as described in the method section. Log2FC of the associated gene was plotted for each of the top, middle and bottom 300 peaks based of their H3K27ac signal change after LPS in Additional file 4 D. However, genes for which log2FC could not be calculated (because one of the FPKM value was 0 which returned a ”-inf” or “+inf” result) were removed. FPKM values in both LPS and Control conditions were used to generate the boxplot in Additional file 2 D.

RT-qPCR

Detroit 562 cells were treated with LPS or fresh medium for 100 minutes (Additional File 10 A and B). Alternatively, the cells were pre-treated with the NF-kB inhibitor or DMSO before LPS stimulation (Figure 4B). Total RNAs were extracted and one microgram of was used for reverse transcription using QuantiTect Reverse Transcription kit (Qiagen) followed by qPCR with LightCycler 480 SYBR Green I Master (Roche) according to the manufacturers’ instructions. The following PCR program was used: 5 minutes at 95°C; 45x(10 seconds at 95°C, 1 minutes at 65°C, 30 seconds at 72°C). The ΔΔCt method was applied to determine the fold change of the genes of interest under LPS compared to the control, normalized with 2 or 3 housekeeping genes (ACTB, GAPDH, TBP). Sequences of the primers used for qPCR are shown in Additional File 11.

ChIP-qPCR

Investigation of H3K27ac mark as well as RELA binding at the *TNF* locus was done by ChIP-qPCR (Figure 4C). The same protocol as the ChIP-seq experiment was used for the chromatin immunoprecipitation except that DNA was eluted after the last wash (before the library on beads preparation) and purified. Eluted DNA was used directly for qPCR, together with input DNA as a control. qPCR was performed with the LightCycler 480 SYBR Green I Master (Roche) according to the manufacturers’ instructions. The following PCR program was followed: 5 minutes at 95°C; 45x(10 seconds at 95°C, 1 minutes at 65°C, 30 seconds at 72°C). The data was analyzed relative to the input to determine the percent of input recovered after immunoprecipitation. Sequences of the primers are shown are shown below.

RT-qPCR	TNF_Forward	CTGCTGCACTTTGGAGTGAT
	TNF_Reverse	CAGCTTGAGGGTTTGCTACA
	ICAM1_Forward	GGGAACAACCGGAAGGTGTA
	ICAM1_Reverse	AGGGTAAGGTTCTTGCCCAC
	ACTB_Forward	AGAGCTACGAGCTGCCTGAC
	ACTB_Reverse	AGCACTGTGTTGGCGTACAG
	GAPDH_Forward	GAAGGTGAAGGTCGGAGTCA
	GAPDH_Reverse	AATTTGCCATGGGTGGAAT
	TBP_Forward	TATAATCCCAAGCGGTTTGC
	TBP_Reverse	GCTGGAAAACCCAACTTCTG
ChIP-qPCR	TNF_H3K27ac_Forward	CCCTTCCATTCTCTCAGTGGG
	TNF_H3K27ac_Reverse	ACTCCTGGGTTCAAGCGATC
	NFKBIA_RELA_Forward	GGAATTTCCAAGCCAGTCAG
	NFKBIA_RELA_Reverse	GGAAGGACTTTCCAGCCACT
	TNF_RELA_Forward	GCCCTGGAGACCCTACATAA
	TNF_RELA_Reverse	AGGATTTTCCCAGCAAGGAT
